# Estimation of the Optimal Number of Replicates in Crop Variety Trials

**DOI:** 10.3389/fpls.2020.590762

**Published:** 2021-01-13

**Authors:** Weikai Yan

**Affiliations:** Ottawa Research and Development Center, Agriculture and Agri-Food Canada, Ottawa, ON, Canada

**Keywords:** crop variety trials, optimal replication, adequate testing, genotype × environment interaction, biplot analysis, heritability

## Abstract

Replicated multi-location yield trials are conducted every year in all regions throughout the world for all regionally important crops. Heritability, i.e., selection accuracy based on variety trials, improves with increased number of replicates. However, each replicate is associated with considerable cost. Therefore, it is important for crop variety trials to be optimally replicated. Based on the theory of quantitative genetics, functions that quantitatively define optimal replication on the single-trial basis and on multi-location trial basis were derived. The function on the single-trial basis often over-estimates the optimum number of replicates; it is the function on multi-location trial basis that is recommended for determining the optimal number of replicates. Applying the latter function to the yield data from the 2015–2019 Ottawa oat registration trials conducted both in Ontario and in other provinces of Canada led to the conclusion that a single replicate or two replicates would have sufficed under the current multi-location trial setup. This conclusion was empirically confirmed by comparing genotypic rankings based on all replicates with that on any two replicates. Use of two replicates can save 33–50% of field plots without affecting the selection efficacy.

## Introduction

Crop variety trials are one of the best funded agronomic studies in the world. Regardless of social and economical levels, crop variety trials are conducted every year in every region for every regionally important crop, to provide information to growers on the performance of existing cultivars and to breeders for releasing new cultivars. Crop variety trials are costly. The cost for growing a single field plot is usually budgeted for $40–50 Canadian dollars in Canada. For a test of 30 entries at 10 locations with four replicates, the cost would be $48000–60000. This amount increases quickly when multiplied with the number of tests, crops, breeding institutes, regions, and years. Therefore, it is of great economical value to minimize the number of plots while maintaining trial efficacy.

Several classical studies investigated the optimum numbers of years, seasons, test locations, and replicates within trials, on the basis of allocating a fixed number of field plots (or available funds), based on the relative magnitude of various variance components, and the relative cost of adding one replicate vs. that adding one location or year (Sprague and Federer, 1951; Hanson and Brim, 1963; Wricke and Weber, 1986; Swallow and Wehner, 1989; Zhou et al., 2011). In reality, most crop variety registration committees require testing for 2 or 3 years for decision making, and there is little space to reduce or increase the number of years. Likewise, mature breeding or regional variety testing programs often have a fixed number of test locations and there is little space to change it. Consequently, changing the number of replicates becomes one of the few options to reduce test cost and/or improve test efficiency. In line with the concept of “adequate testing” (Yan, 2016), this study deals with “optimal replication” in crop variety trials.

Oat variety trials in Canada will be used as an example. The oat (*Avena satia* L.) breeding program in the Ottawa Research and Development Center (ORDC), Agriculture and Agri-Food Canada (AAFC) is based in Ottawa, Ontario; it has the mandate to develop new oat cultivars for eastern Canada, including Ontario, Quebec, and the Maritime provinces. To fulfill this task, we conduct yearly oat variety registration trials at multiple locations in Ontario and across Canada; the locations are chosen for necessity or accessibility. While four replicates are required by the Ontario Cereal Crops Committee, three are required by other Canadian variety registration supporting committees. So, our registration trials are conducted with four replicates at locations in Ontario and three at locations in other provinces. On the other hand, in most crop breeding programs it is common to conduct a two-rep or even a single-rep test due to a great number of entries or limited field space or other resources. For example, we conduct 2-rep yield trials at three locations at an early breeding stage and the data appear to be useful for both conventional selection and genomic model development (Yan et al., 2019). The purpose of this study was to develop theoretical functions that quantitatively define “optimal replication” and to investigate whether two replicates are adequate for reliable oat variety evaluation in Canada.

## Materials and Methods

### Theory Development on Optimal Replication

#### Optimal Replication on the Single-Trial Basis

The achieved heritability within a single trial (*H*_*ST*_) is defined by Falconer (1989); DeLacy et al. (1996):

(1) $$H_{S\,T}=\frac{\sigma_{G}^{2}}{\sigma_{G}^{2}+\frac{\sigma_{\varepsilon }^{2}}{r}},$$

where $\sigma_{G}^{2}$ is the genotypic variance and $\sigma_{\varepsilon }^{2}$ the experimental error variance estimated on a single trial basis, and *r* is the number of replicates in the trial. This equation indicates that for a given set of genotypes, natural conditions, and management, the trial heritability can be improved only by increasing the number of replicates.

From Eq. 1, we have

(2) $$r=\left(\frac{H_{S\,T}}{1-H_{S\,T}}\right)\,\left(\frac{\sigma_{\varepsilon }^{2}}{\sigma_{G}^{2}}\right).$$

Observing the curvilinear relationship between *r* and *H*_*ST*_ revealed that heritability improves nearly linearly with the increase in *r* when the heritability is lower than a certain level, say, 0.75, and the effect of increasing *r* gradually diminishes after that (Yan et al., 2015). Therefore, a trial may be regarded as optimally replicated when the achieved heritability is equal to 0.75, and the number of replicates required to achieve this level of accuracy can be estimated by Yan et al. (2015):

(3) $$r_{H_{S\,T}=0.75}=\text{max}\,\left(1,3\,\left(\frac{\sigma_{\varepsilon }^{2}}{\sigma_{G}^{2}}\right)\right).$$

The so-estimated number of replicates may be referred as the number of replicates for optimal replication. Equation 3 was adopted to estimate the required number of replicates for China national cotton (Xu et al., 2016) and wheat (Zhang et al., 2020) variety trials, the required number of replicates for wheat and cotton variety trials in the Mediterranean regions (Baxevanos et al., 2017a, b), the required number of replicates in soybean variety trials in Brazil (Woyann et al., 2020), and the required number of replicates for winter wheat in California (George and Lundy, 2019).

#### Optimal Replication on the Basis of Single-Year, Multi-Location Test

The heritability on the basis of single-year, multi-location test is determined by

(4) $$H_{M\,L}=\frac{\sigma_{G,M\,L}^{2}}{\sigma_{G,M\,L}^{2}+\frac{\sigma_{G\,L}^{2}}{l}+\frac{\sigma_{\varepsilon ,M\,L}^{2}}{l\,r}},$$

where $\sigma_{G,M\,L}^{2}$ is the genotypic variance and $\sigma_{\varepsilon ,M\,L}^{2}$ the experimental error variance estimated on the single-year, multi-location trial basis; $\sigma_{G\,L}^{2}$ is the variance for genotype by location interaction (GL) and *l* is the number of locations. From Eq. 4, the number of replicates required to achieve a target level of heritability on the multi-location basis is determined by:

(5) $$r=\left(\frac{\sigma_{\varepsilon ,M\,L}^{2}}{l\,\sigma_{G,M\,L}^{2}}\right)\,\left(\frac{H_{M\,L}}{1-H_{M\,L}/H_{M\,M\,L}}\right),$$

where *H*_*MML*_ is the maximum achievable across-location heritability and is determined by:

(6) $$H_{M\,M\,L}=\frac{\sigma_{G,M\,L}^{2}}{\sigma_{G,M\,L}^{2}+\frac{\sigma_{G\,L}^{2}}{l}},$$

Equation 6 is a special case of Eq. 4, i.e., the cross-location heritability with 0 experimental error variance or with an infinite number of replicates. The target level of cross-location heritability must be smaller than the maximum possible heritability, i.e., *H*_*ML*_¡*H*_*MML*_, for Eq. 5 to be meaningful. Therefore, the target cross-location heritability should not be a specific level of *H*_*ML*_; rather, it should be a certain level of *H*_*ML*_/*H*_*MML*_, which may be called “relative cross-location heritability.” The relative cross-location heritability is the measure for adequate replication in the multi-location trial framework. If the target cross-location heritability is set such that *H*_*ML*_/*H*_*MML*_=0.75, i.e., *H*_*ML*_=0.75*H*_*MML*_, then Eq. 5 becomes

(7) $$r_{H_{M\,L}=0.75\,H_{M\,M\,L}}=\left(1,3\,\left(\frac{\sigma_{\varepsilon ,M\,L}^{2}}{l\,\sigma_{G,M\,L}^{2}}\right)\,H_{M\,M\,L}\right).$$

Because the error variance across locations, $\sigma_{\varepsilon ,M\,L}^{2}$, is the accumulative error variance of individual locations, σ^2^, the value of $\frac{\sigma_{\varepsilon ,M\,L}^{2}}{l\,\sigma_{G,M\,L}^{2}}$ in Eq. 7 should be similar to that of $\frac{\sigma_{\varepsilon }^{2}}{\sigma_{G}^{2}}$ in Eq. 3; the optimal number of replicates estimated based on Eq. 7, therefore, shrinks with *H*_*MML*_, relative to that based on Eq. 3. The smaller the *H*_*MML*_, the greater the shrinkage is, and the fewer replicates will be needed. This may appear counter intuitive. However, it means that when *H*_*MML*_ is low, the key to improve the cross-location heritability is not to increase the number of replicates; rather, it is to increase the number of locations or to divide the region into meaningful mega-environments.

From Eq. 6 and analogous to Eq. 3, the required number of locations for adequate testing, i.e., to achieve *H*_*MML*_=0.75 in a mega-environment, can be estimated by Yan et al. (2015)

(8) $$l_{H_{M\,M\,L}=0.75}=\text{max}\,\left(1,3\,\left(\frac{\sigma_{G\,L}^{2}}{\sigma_{G,M\,L}^{2}}\right)\right).$$

More locations would be needed if *H*_*MML*_¡0.75, and fewer locations would be needed if *H*_*MML*_¿0.75, relative to the actual number of locations used in the test.

#### Optimal Replication on the Basis of the Multi-Year, Multi-Location Test

The discussion above can be extended to multi-year, multi-location tests. The heritability under the multi-year, multi-location framework, *H*_*MLY*_, is determined by

(9) $$H_{M\,L\,Y}=\frac{\sigma_{G,M\,L\,Y}^{2}}{\sigma_{G,M\,L\,Y}^{2}+\frac{\sigma_{G\,L}^{2}}{l}+\frac{\sigma_{G\,Y}^{2}}{y}+\frac{\sigma_{G\,L\,Y}^{2}}{l\,y}+\frac{\sigma_{\varepsilon ,M\,L\,Y}^{2}}{l\,y\,r}},$$

Where $\sigma_{G,M\,L\,Y}^{2}$ is the genotypic variance and $\sigma_{\varepsilon ,M\,L\,Y}^{2}$ the experimental error variance estimated on the multi-location, multi-year trial basis; $\sigma_{G\,Y}^{2}$ and $\sigma_{G\,L\,Y}^{2}$ are variances for genotype by year interaction (GY) and genotype by location by year three-way interaction (GLY), respectively, and *y* is the number of years the test is conducted.

From Eq. 9 the number of replicates required to achieve a certain level of heritability is determined by:

(10) $$r=\left(\frac{\sigma_{\varepsilon ,M\,L\,Y}^{2}}{l\,y\,\sigma_{G,M\,L\,Y}^{2}}\right)\,\left(\frac{H_{M\,L\,Y}}{1-H_{M\,L\,Y}/H_{M\,M\,L\,Y}}\right),$$

where

(11) $$H_{M\,M\,L\,Y}=\frac{\sigma_{G,M\,L\,Y}^{2}}{\sigma_{G,M\,L\,Y}^{2}+\frac{\sigma_{G\,L}^{2}}{l}+\frac{\sigma_{G\,Y}^{2}}{y}+\frac{\sigma_{G\,L\,Y}^{2}}{l\,y}}$$

*H*_*MMLY*_ is the maximum possible heritability on the multi-year, multi-location basis. Equation 11 is a special case of Eq. 9, i.e., the cross location and year heritability, assuming 0 experimental error variance or infinite number of replicates. If the target heritability is set to *H*_*MLY*_=0.75*H*_*MMLY*_, then the number of replicates for optimal replication can be determined by

(12) $$r_{H_{M\,L\,Y}=0.75\,H_{M\,M\,L\,Y}}=\left(1,3\,\left(\frac{\sigma_{\varepsilon ,M\,L\,Y}^{2}}{l\,y\,\sigma_{G,M\,L\,Y}^{2}}\right)\,H_{M\,M\,L\,Y}\right).$$

The implications discussed above regarding Eq. 7 can also be extended to Eq. 12. Furthermore, the definition of heritability in Eq. 9 is for variety trial systems in which a single crop is grown each year and is under a single management. Factors such as season and management should be added when multiple crops are grown in the same year (e.g., Swallow and Wehner, 1989) or in agronomic experiments in which multiple managements are involved.

Note that the definition of the heritability at various levels is consistent with the concept of mixed effect models (DeLacy et al., 1996). The genotypic main effect (G), the genotype by environment effects (GL, GY, GLY), and the experimental errors (ε) are treated as random effects as they appear in the formulas of heritability (Eqs 1, 4, and 9). Effects not included in the heritability formulas, such as the main effects of block, location, and year, are treated as fixed effects.

#### The Sample Data Used in This Study

The yield data from the 2015 to 2019 ORDC oat registration test were used as an example in this study (the raw data, which belong to AAFC, are available upon request). Each year the test was conducted at several locations within Ontario as well as in other provinces, including Quebec and Prince Edward Island in eastern Canada and Manitoba and Alberta in western Canada, as listed in Table 1. The trials were conducted with four replicates at the Ontario locations, as required by the Ontario Cereal Crops Committee, and three at locations in other provinces. Each year the same set of 36 oat genotypes were tested at all locations, and the set of genotypes varied each year. All trials were conducted based on randomized complete blocks designs and in rain-fed conditions. Not all locations listed in Table 1 were used in all years. Locations in Ontario have generally lower latitudes (<45.5°N), except for the northern Ontario location New Liskeard (Table 1).

**TABLE 1:** Test locations involved in the 2015–2019 oat registration test and their geographical coordinates (sorted by latitude).

Location code	Location names	Latitude (°N)	Longitude (L°)	Number of replicates
ELORA	Elora, ON	43.7	−80.4	4
PALM	Palmerston, ON	43.8	−80.8	4
DUN	Dundalk, ON	44.2	−80.4	4
HECK	Heckston, ON	45.0	−75.5	4
OTT	Ottawa, ON	45.4	−75.7	4
PRIN2	Princeville, QC	46.2	−71.9	3
HAR	Harrington, PE	46.4	−63.2	3
LAPO3	La Poctiere, QC	47.4	−70.2	3
NL	New Liskeard, ON	47.5	−79.7	4
NORM3	Normandin, QC	48.8	−72.6	3
BRA	Brandon, MB	49.9	−99.9	3
LAC	Lacombe, AB	52.5	−113.8	3

### GGE Biplot Analysis for Cross-Location Genotype Evaluation Based on All Replicates vs. Any Two Replicates

Multi-location trial data analysis may be conducted in two steps: the first is single trial data analysis, including adjusting for any block effects and within-block spatial variation, and the second is cross-location analysis for making selection decisions (Yan, 2014). Data from multi-location trials within a year are usually balanced by design and are therefore most informative; this was the strategy used in this study. GGE (genotypic main effect, G, plus genotype by environment interaction, GE) biplot analysis is a graphical and informative approach to multi-location data analysis (Yan, 2014). Yearly multi-location data were analyzed using GGE biplots to visualize if the G + GE pattern based on all replicates can be sufficiently approximated by that based on any two of the replicates. In particular, GGE biplots were used to visualize whether the highest yielding genotype(s) identified based on all (four in Ontario trials and three in non-Ontario trials) replicates can also be identified by using any two of the replicates. Correlation coefficients were calculated to show how well the genotypic means based on all replicates were approximated by that from any two replicates. The analysis was conducted separately for Ontario trials and non-Ontario trials, because they were conducted with different number of replicates. The analyses were conducted using the GGEbiplot software (Yan, 2001, 2014).

## Results

### Number of Replicates for Optimum Replication on the Single Trial Basis

The estimated trial heritability (Eq. 1), the optimum number of replicates for each trial (Eq. 3), the mean trial heritability, and the mean required number of replicates averaged across locations each year are presented in Table 2; results for trials within Ontario and those in other provinces are analyzed and presented separately.

**TABLE 2:** Achieved trial heritability and estimated number of replicates to achieve a trial heritability of 0.75 for each of the trials.

Year	Number of locations	Achieved trial heritability		Estimated optimum number of replicates	
		Range	Mean	Range	Mean
**Trials in Ontario**					
2015	4	0.47–0.89	0.77	1.9–13.3	4.95
2016	5	0.70–0.94	0.79	1.0–5.1	3.38
2017	5	0.58–0.87	0.80	1.8–8.7	3.72
2018	5	0.66–0.84	0.76	2.3–6.1	3.94
2019	5	0.78–0.95	0.91	1.0–3.4	1.54
**Non-Ontario trials**					
2015	6	0.36–0.96	0.86	1.0–15.8	1.75
2016	5	0.70–0.91	0.82	1.0–3.8	2.05
2017	4	0.52–0.85	0.65	1.6–8.3	5.40
2018	4	0.0–0.89	<0.62^a^	1.1–3.2	>1.90^a^
2019	4	0.0–0.70	<0.50^a^	3.8–5.1	>4.63^a^

*^*a*^The trial heritability was not estimable for the trials at Brandon in 2018 and at Lacombe in 2019; these trials were excluded from calculating the cross-location means.*

For the Ontario trials, the estimated optimum number of replicates on the single trial basis varied greatly from location to location and from year to year. The mean across locations ranged from 1.54 to 4.95, depending on the year (Table 2, upper part). The overall mean across years was around 4.0, which was the number of replicates actually used in these trials.

For the non-Ontario trials, the achieved trial heritability, hence, the estimated optimum number of replicates also varied greatly, depending on the location and year. The Brandon 2018 trial had a near 0 genotypic variance and hence a near 0 heritability, with a trial coefficient of variation (CV) of 22.8%. The Lacombe 2019 trial also had a near 0 heritability with a CV of 10.4%. CV is a measure of trial accuracy independent of genotypic variation while heritability is a measure of both trial accuracy and trial usefulness to genotype valuation (Yan, 2014). These two trials were therefore considered as failed and were excluded from calculating the yearly means. The mean estimated optimum number of replicates averaged across all locations was from 1.75 to 5.40 for 2015, 2006, and 2017 (Table 2, lower part). The average across years was 3.0, which was the number of replicates actually used.

In summary, the analysis on the single trial basis did not support the hypothesis that two replicates would have sufficed for reliable genotype evaluation.

### Estimated Optimum Number of Replicates on the Multi-Location Basis

For the Ontario trials, the estimated optimum number of replicates on the multi-location basis was around 1.0 for years 2015–2018 and it was only 0.2 for 2019 (*r*_*ML*_, Table 3, upper part). These values sharply contrast with that estimated on the single trial basis (Table 2).

**TABLE 3:** The ratio of genotype by location interaction variance over genotypic variance ($\sigma_{G\,L}^{2}/\sigma_{G}^{2}$),the ratio of experimental error variance over genotypic variance ($\sigma^{2}/\sigma_{G}^{2}$), the estimated optimum number of replicates on the multi-location basis (*r*_*ML*_), and estimated number of locations for adequate testing(*l*_*HML*_) for theOntario and non-Ontario tests in individual years.

Year	Number oflocations	$\sigma_{\text{GL}}^{2}/\sigma_{G}^{2}$	$\sigma_{\varepsilon }^{2}/\sigma_{G}^{2}$	H_ML_(Eq. 4)	H_MML_(Eq. 6)	r_ML_(Eq. 7)	l_H_ML__(Eq. 8)
**Ontario trials**							
2015	4	2.23	2.36	0.59	0.64	1.1	6.7
2016	5	1.83	2.25	0.68	0.73	1.0	5.5
2017	5	3.14	3.26	0.56	0.61	1.2	9.4
2018	5	0.87	2.25	0.78	0.85	1.1	2.6
2019	5	1.00	0.48	0.82	0.83	0.2	3.0
**Non-Ontario trials**							
2015	6	2.78	3.44	0.62	0.68	1.2	8.3
2016	6	2.36	1.93	0.67	0.72	0.7	7.1
2017	4	3.50	6.13	0.42	0.53	2.5	10.5
2018	4	8.99	40.96	0.15	0.31	9.5	27.0
2019	4	0.55	4.79	0.65	0.88	3.2	1.6
2018-BRA^a^	3	9.82	10.15	0.82	0.23	2.4	29.4
2019-LAC^b^	3	0.76	2.78	0.82	0.80	2.2	2.3

*2018-BRA^*a*^ refers to the 2018 non-Ontario test excluding the Brandon trial, which had a 0 heritability. 2019-LAC^*b*^ means the 2019 non-Ontario test excluding the Lacombe trial, which had a 0 heritability.*

For the non-Ontario trials, the estimated optimum number of replicates varied greatly, ranging from 0.7 for 2016 to 9.5 for 2018 (*r*_*ML*_, Table 3, lower part). The high estimated number for 2018 was due to its high experimental error, 40 times higher than the genotypic variance. This was due to the extremely poor data quality at the Brandon location. When data from this trial were excluded, the estimated optimum number of replicates was reduced to 2.4 (“2018-BRA” in Table 3). Similarly, the relatively high number of replicates estimated for 2019 (3.2, Table 3) was due to poor data quality at the Lacombe location. When data from this location was excluded, the number was reduced to 2.2 (“2019-LAC,” Table 3). Therefore, the results for the non-Ontario tests indicate that from 0.7 to 2.5, averaged 1.8, replicates were required for optimal replication. In summary, the cross-location analysis supported the hypothesis that two replicates would suffice for reliable genotype evaluation.

Also presented in Table 3 is the estimated number of test locations required for “adequate testing,” which was greater than the actual number of locations used in three of the 5 years for the Ontario tests (2015, 2016, and 2017) and much greater in four of the 5 years for the non-Ontario tests (2015–2018). This suggests that increasing the number of test locations will be effective in improving the cross-location heritability and therefore selection accuracy. Alternatively, it suggests that multiple mega-environments were involved in the trials. Indeed, it is known that New Liskeard (northern Ontario) and the other Ontario locations belong to contrasting mega-environments (Yan et al., 2015, 2020). It is also known that the locations in western Canada (Brandon in MB and Lacombe in AB) and those in eastern Canada (Normandin, Princeville, and la Poctiere in QC and Harrington in PE) belong to different mega-environments (Yan et al., 2020).

### Cross-Location Genotypic Ranking Based on All Replicates vs. Any Two Replicates

#### The 2019 Ontario Trials

Relatively high trial heritability was achieved at all five Ontario locations in 2019 (ranged from 0.78 to 0.95 and averaged 0.91). As a result, the estimated optimum number of replicates on the single trial basis was small, ranged from 1.0 to 3.4 and average 1.54 (Table 2). This means that on average 1.54 replicates would have sufficed for reliable genotype evaluation. The optimum number of replicates on a multi-location basis was estimated to be 1.1 (Table 3), indicating that a single replicate would suffice. Therefore, for this dataset it is expected that genotypic evaluation based on any two replicates would sufficiently approximate that based on all replicates.

**FIGURE 1:**
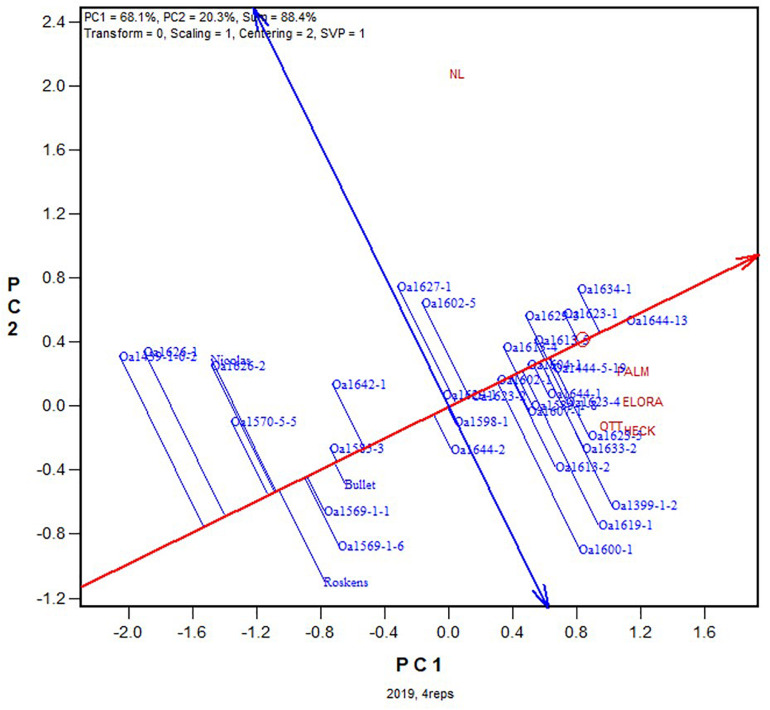
GGE biplot based on the yield data from all four replicates for the 2019 Ontario trials. See Table 1 for the full names of the locations.

Presented in Figure 1 is the GGE biplot that approximately displays the 2019 yield data for each of the tested genotypes at each of the five locations across all four replicates. The following patterns can be seen from this biplot. (1) The five locations fell into two groups: NL (northern Ontario) was clearly separated from the other four locations OTT, HECK, ELORA, and PALM (southern or eastern Ontario), consistent with the conclusion from a long-term study that northern Ontario and southern Ontario are two contrasting mega-environments (Yan et al., 2015, 2020). (2) The highest yielding genotype was OA1644-13, followed by OA1634-1 and a group of other genotypes. The red line with a single arrow is the average environment axis (AEA), with the arrow pointing to higher mean yield across locations. Although a biplot does not have a measure of uncertainty, the magnitude of the difference between two genotypes can be visually assessed by the distance between them. (3) Some crown rust resistant genotypes, including OA1399-1-2, OA1600-1, and OA1619-1, yielded well at the crown rust prone locations OTT, HECK, ELORA, and PALM but poorly at NL where crown rust rarely occurs.

**FIGURE 2:**
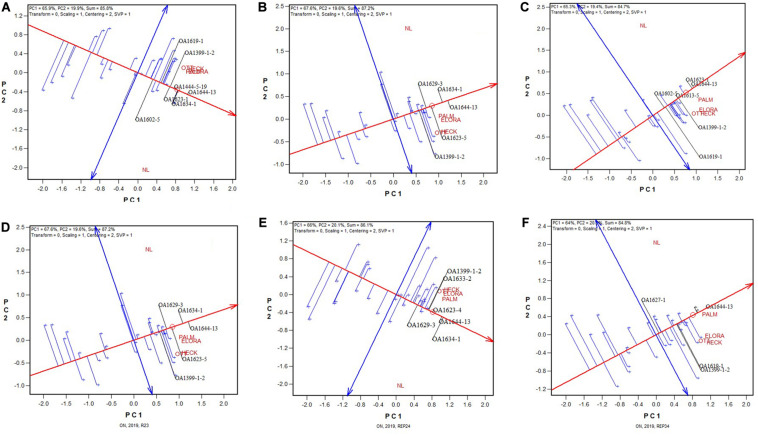
GGE biplots based on the yield data from six 2-rep combinations for the 2019 Ontario trials; **(A)** combination of replications 1 and 2; **(B)** combination of replications 1 and 3; **(C)** combination of replications 1 and 4; **(D**) combination of replications 2 and 3; **(E)** combination of replications 2 and 4; and **(F)** combination of replications 3 and 4. Note that these 2-rep combinations are random samples of all possible 2-rep combinations because replication 1 at one location was unrelated to replication 1 at other locations. See Table 1 for the full names of the test locations. Some genotypes are spelled out in black color while most of them are represented by “+” for clarity.

Presented in Figure 2 are six GGE biplots based on any two of the four replicates. Note that each of the 2-rep combinations represented in the biplots represents a random sample of all possible 2-rep combinations, because the replicates were nested within test locations and the replicate labeled “1” at one location was unrelated to that at any other location. It can be seen that the patterns observed from Figure 1 remained largely true in each of the six biplots in Figure 2. This indicates that two replicates would have sufficed for the 2019 Ontario test.

**FIGURE 3:**
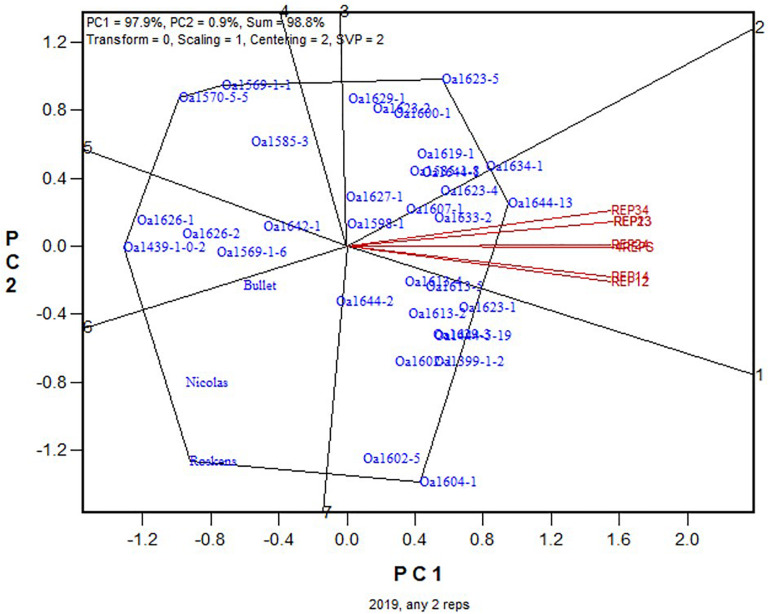
Summary biplot for the 2019 Ontario trials to show the similarity/dissimilarity between genotypic rankings based on any 2-rep combinations and that based on all four replicates. 4REPS, genotypic ranking based on all four replicates; REP12, genotypic ranking based on replicates 1 and 2; REP13, genotypic ranking based on replicates 1 and 3, and so on.

For further elucidation, the predicted mean yield for each genotype based on all replicates (Figure 1) and that based on each of the six 2-repcombinations (Figure 2) are presented in Table 4. This table is graphically displayed in the biplot in Figure 3, which serves as a concise summary of the seven biplots in Figures 1, 2. The following can be seen from Figure 3. (1)The genotypic rankings from the seven biplots were closely correlated, as indicated by the acute angles among the vectors. The cosine of the angle between any two vectors approximates the Pearson correlation between them; this can be verified from the correlation coefficients presented in Table 5. (2) All six 2-rep combinations (REP12, REP13, REP14, REP23, REP24, and REP34) as well as the full dataset (4REPS) identified OA1644-13 as the highest yielding genotype, as they all fell in the OA1644-13 sector defined between the two radiate lines labeled “1” and “2.” Hereafter, summary biplots like Figure 3 will be used to compare genotypic rankings based on all replicates vs. that based on any two of the replicates for the other datasets.

**TABLE 4:** Mean yield (kg ha^–1^) across locations based on all or any two of the four replicates for the 2019 Ontario test.

Genotype	4REPS^a^	REP12	REP13	REP14	REP23	REP24	REP34
Bullet	3259	3432	3310	3200	3310	3217	3093
Nicolas	2934	3029	2756	3058	2756	2915	2784
OA1399-1-2	4402	4524	4425	4431	4425	4546	4302
OA1439-1-0-2	2534	2511	2407	2595	2407	2633	2481
OA1444-5-19	4443	4596	4501	4380	4501	4329	4306
OA1569-1-1	3106	2978	3152	3069	3152	3156	3248
OA1569-1-6	3094	3048	2984	3205	2984	3135	3138
OA1570-5-5	2886	2736	2853	2858	2853	2780	3017
OA1585-1-8	4263	4161	4327	4226	4327	4324	4367
OA1585-3	3272	3263	3366	3161	3366	3437	3269
OA1598-1	3887	3869	3917	3879	3917	3909	3914
OA1600-1	4163	4214	4425	3954	4425	4021	4164
OA1602-1	4172	4259	4127	4183	4127	4417	4054
OA1602-5	4004	4201	3901	4114	3901	3979	3828
OA1604-1	4335	4680	4369	4297	4369	4384	3991
OA1607-1	4238	4156	4265	4231	4265	4369	4319
OA1613-2	4237	4316	4247	4254	4247	4404	4212
OA1613-4	4268	4326	4301	4255	4301	4066	4244
OA1613-5	4421	4427	4415	4419	4415	4180	4411
OA1619-1	4301	4351	4512	4139	4512	4224	4314
OA1623-1	4613	4516	4484	4734	4484	4494	4746
OA1623-2	4020	4054	4279	3789	4279	4076	3981
OA1623-4	4430	4408	4552	4322	4552	4625	4453
OA1623-5	4452	4389	4668	4247	4668	4469	4559
OA1626-1	2651	2597	2530	2699	2530	2511	2637
OA1626-2	2925	2716	2667	3114	2667	2910	3073
OA1627-1	3929	3892	3961	3856	3961	3710	3936
OA1629-1	3890	3765	4001	3778	4001	3946	3986
OA1629-3	4434	4676	4571	4290	4571	4316	4216
OA1633-2	4399	4380	4468	4353	4468	4540	4459
OA1634-1	4728	4672	4892	4563	4892	4842	4734
OA1642-1	3423	3350	3347	3482	3347	3183	3472
OA1644-1	4362	4383	4463	4202	4463	4249	4425
OA1644-13	4878	4853	5002	4775	5002	4814	4934
OA1644-2	3807	3950	3863	3751	3863	3888	3666
Roskens	2950	3138	2754	3171	2754	2843	2777

*^*a*^4REPS, mean yield based on all four replicates; REP12, mean yield based on replicates 1 and 2; REP13, mean yield based on replicates 1 and 3, and so on. Note that the replicates are nested within locations such that the replicate labeled “1” at one location was unrelated to that in other locations.*

**TABLE 5:** Pearson correlation coefficients between genotypic means based on all or any two of the four replicates for the 2019 Ontario test.

	4REPS^a^	REP12	REP13	REP14	REP23	REP24
REP12	0.985					
REP13	0.991	0.975				
REP14	0.988	0.976	0.958			
REP23	0.991	0.975	1.000	0.958		
REP24	0.981	0.966	0.975	0.968	0.975	
REP34	0.985	0.941	0.978	0.969	0.978	0.965

*^*a*^4REPS, mean yield based on all four replicates; REP12, mean yield based on replicates 1 and 2; REP13, mean yield based on replicates 1 and 3, and so on. Note that the replicates are nested within locations such that the replicate labeled “1” at one location was unrelated to that in other locations.*

#### The Ontario Trials in Years 2015–2018

The same analyses as described for the 2019 Ontario dataset were conducted for the 2015–2018 datasets. The trial heritability for 2015–2018 Ontario tests was considerably lower than that in 2019 (Table 2). As a result, the estimated optimum number of replicates was larger, ranging from 3.72 in 2017 to 4.95 in 2015 (Table 2). On the multi-location basis, however, it was estimated that a single replicate would suffice for reliable genotype evaluation for all these datasets (Table 3). It would be interesting to see if two replicates would still have sufficed in these years, particularly in 2015, which had the lowest trial heritability.

**FIGURE 4:**
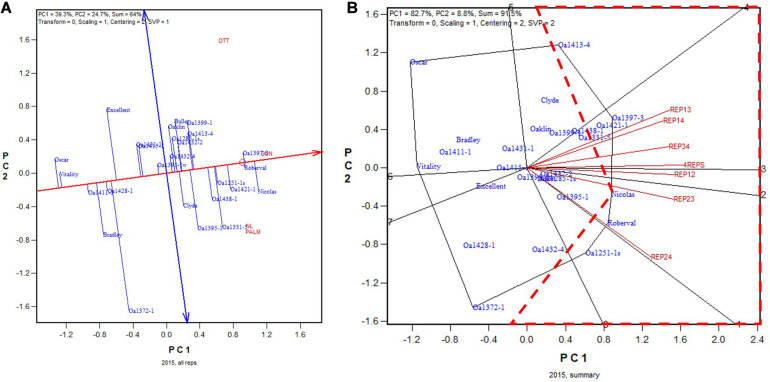
Biplots for the 2015 Ontario trials. **(A)** GGE biplot to show genotype ranking based on data from all four replicates, and **(B)** summary biplot to show the genotype rankings based on all four replicates and on any two of them. 4REPS, genotypic ranking based on all four replicates; REP12, genotypic ranking based on replicates 1 and 2; REP13, genotypic ranking based on replicates 1 and 3, and so on.

The GGE biplot and the summary biplot for the 2015 Ontario dataset are presented in Figures 4A,B, respectively. The GGE biplot identified the highest yielding genotypes to be Nicolas, followed by Roberval and OA1397-3, and then by a group of others (Figure 4A). Due to the relatively poor trial heritability (Table 2), the correlations among the six 2-rep combinations in genotype ranking were relatively low, as indicated by the relatively wide angles between them (Figure 4B). The correlation coefficient between genotypic ranking based on all four replicates and that based on the six2-rep combinations ranged from 0.78 to 0.94 and averaged 0.89 (*n* = 36). The highest yielding genotype identified on all four replicates, Nicolas (Figure 4A), was identified as the highest yielding only in REP12 although it was close to be the highest yielding in other 2-rep combinations (Figure 4B). In the worst cases, Nicolas was the 4th highest yielding in REP13 (after OA1397-3, OA1421-1, and OA1413-4) and the 3rd in REP24 (after Roberval and OA1251-1s) (Figure 4B). In other words, the highest yielding genotype identified based on all four replicates was at least the 4th highest yielding if the trials were conducted with only two replicates. Nicolas has been a popular cultivar in the northern regions of eastern Canada (Yan et al., 2016, 2019). Because multiple genotypes are advanced each year, it can be concluded that two replicates would have sufficed for the 2015 test even though it had relatively low single trial heritability. The frame of broken lines in Figure 4B was defined by two straight lines that started from the placement of Nicolas and were perpendicular to the two 2-rep vectors that had the widest angle (REP13 and REP24). Genotypes within this frame are predicted to be higher yielding than Nicolas in REP13 or REP24.

**FIGURE 5:**
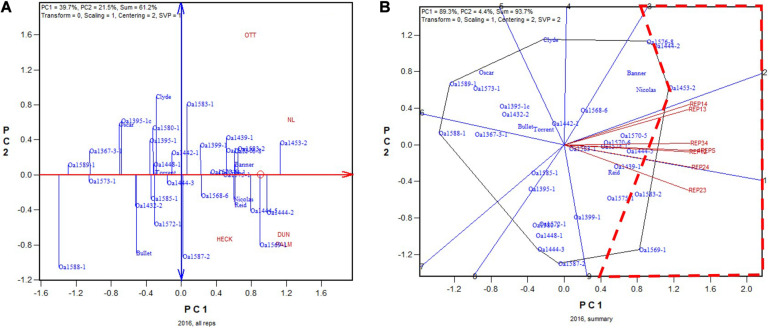
Biplots for the 2016 Ontario trials. **(A)** GGE biplot to show genotype ranking based on data from all four replicates, and **(B)** summary biplot to show the genotype rankings based on all four replicates and on any two of them. 4REPS, genotypic ranking based on all four replicates; REP12, genotypic ranking based on replicates 1 and 2; REP13, genotypic ranking based on replicates 1 and 3, and so on.

In 2016, OA1453-2 (registered as AAC Stature in 2020) was identified as the highest yielding genotype based on all four replicates (Figure 5A). It was identified as the highest yielding genotype in all 2-rep combinations except REP23, in which it was the 2nd or 3rd highest yielding, after OA1569-1 and possibly also OA1583-2 (Figure 5B). The correlation between genotypic means based on all four replicates and that based on any two replicates ranged from 0.91 to 0.94 and averaged 0.93 (*n* = 36).

**FIGURE 6:**
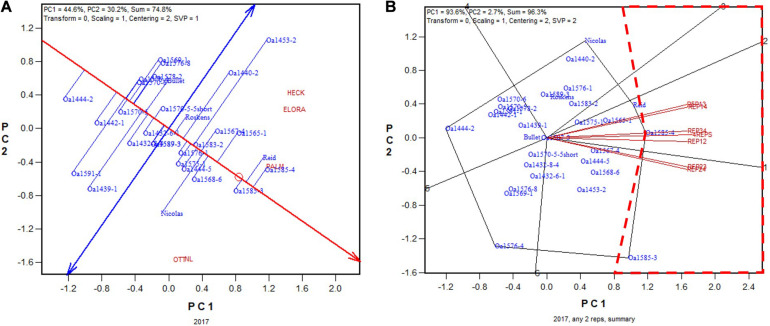
Biplots for the 2017 Ontario trials. **(A)** GGE biplot to show genotype ranking based on data from all four replicates, and **(B)** summary biplot to show the genotype rankings based on all four replicates and on any two of them. 4REPS, genotypic ranking based on all four replicates; REP12, genotypic ranking based on replicates 1 and 2; REP13, genotypic ranking based on replicates 1 and 3, and so on.

In 2017, the highest yielding genotypes based on all four replicates was OA1585-4, closely followed by Reid and OA1585-3 (Figure 6A). OA1585-4 was identified as the highest yielding for four of the six 2-rep combinations, and it was the 2nd, after OA1585-3, for the other two, REP23 and REP24 (Figure 6B). The correlation between genotypic means based on all four replicates and that based on any two replicates ranged from 0.96 to 0.97 and averaged 0.96 (*n* = 36).

**FIGURE 7:**
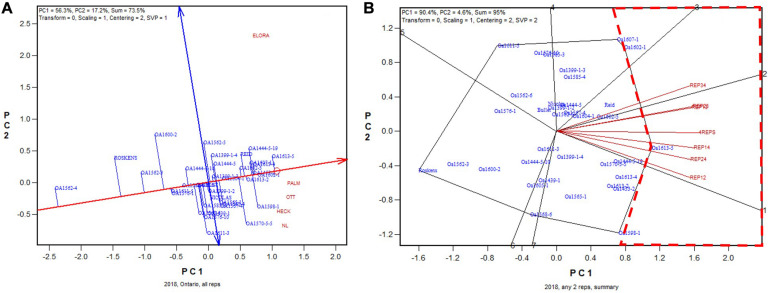
Biplots for the 2018 Ontario trials. **(A)** GGE biplot to show genotype ranking based on data from all four replicates, and **(B)** summary biplot to show the genotype rankings based on all four replicates and on any two of them. 4REPS, genotypic ranking based on all four replicates; REP12, genotypic ranking based on replicates 1 and 2; REP13, genotypic ranking based on replicates 1 and 3, and so on.

In 2018, the highest yielding genotype was identified to be OA1613-5 (to be released in January 2021) based on all replicates (Figure 7A). This genotype was identified to be the highest yielding for five of the six 2-rep combinations, and it was close to the highest yielding (similar to OA1602-1) for the other 2-rep combination, REP34 (Figure 7B). The correlation between genotypic means based on all four replicates and that based on any two replicates ranged from 0.92 to 0.96 and averaged 0.94 (*n* = 36). In summary, for all 5 years, two replicates were proven to be adequate for reliable genotype evaluation for the Ontario tests.

#### The Non-Ontario Tests

**FIGURE 8:**
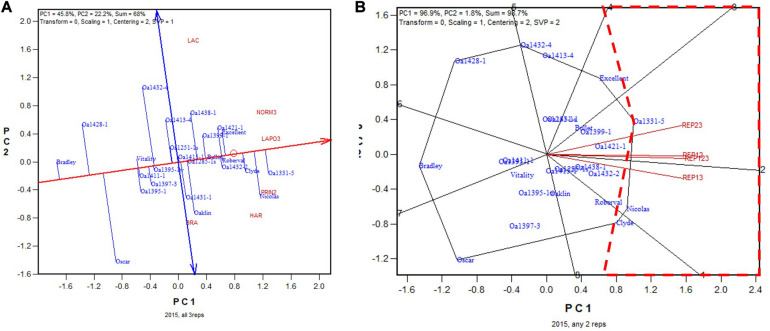
Biplots for the 2015 non-Ontario trials. **(A)** GGE biplot to show genotype ranking based on data from all three replicates, and **(B)** summary biplot to show the genotype rankings based on all three replicates and on any two of them. REP123, genotypic ranking based on all three replicates; REP12, genotype ranking based on replicates 1 and 2; REP13, genotype ranking based on replicates 1 and 3.

The non-Ontario trials were conducted with three replicates. In the 2015 test, the highest yielding genotype, based on all three replicates, was OA1331-5, followed by Nicolas and Clyde (Figure 8A). OA1331-5 was identified as the highest yielding in two of the three 2-rep combinations, and for REP13, it was the 3rd highest yielding, after Nicolas and Clyde (Figure 8B). Nicolas was a selection from OA1331-5 (Yan et al., 2016). Thus, any two replicates would have sufficed to identify the highest yielding genotypes for this test. The correlation between genotypic means based on all three replicates and that based on any two replicates ranged from 0.97 to 0.99 and averaged 0.98 (*n* = 36).

**FIGURE 9:**
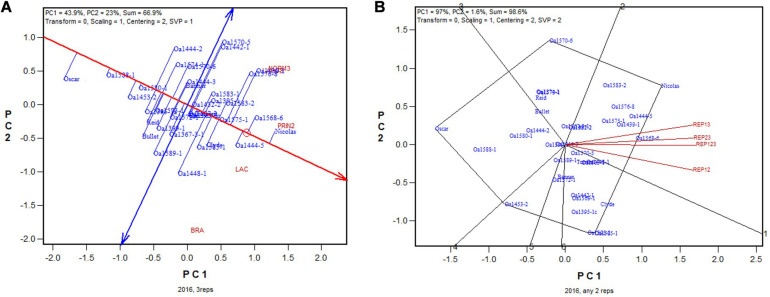
Biplots for the 2016 non-Ontario trials. **(A)** GGE biplot to show genotype ranking based on data from all three replicates, and **(B)** summary biplot to show the genotype rankings based on all three replicates and on any two of them. REP123, genotypic ranking based on all three replicates; REP12, genotype ranking based on replicates 1 and 2; REP13, genotype ranking based on replicates 1 and 3.

In 2016 the highest yielding genotypes, identified on all three replicates, was Nicolas (Figure 9A), which was also identified as the highest yielding in all three 2-rep combinations (Figure 9B). The correlation between genotypic means based on all three replicates and that based on any two replicates ranged from 0.94 to 0.98 and averaged 0.96 (*n* = 36).

**FIGURE 10:**
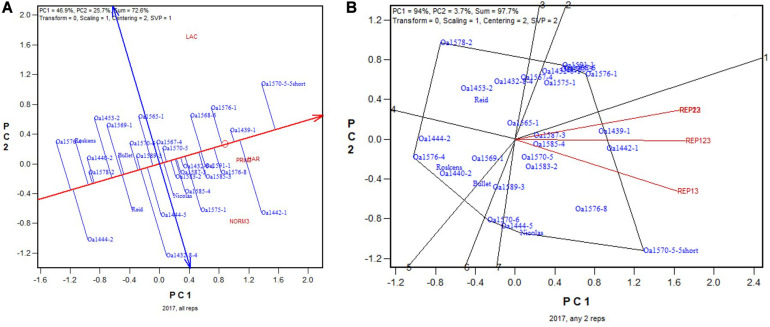
Biplots for the 2017 non-Ontario trials. **(A**) GGE biplot to show genotype ranking based on data from all three replicates, and **(B)** summary biplot to show the genotype rankings based on all three replicates and on any two of them. REP123, genotypic ranking based on all three replicates; REP12, genotype ranking based on replicates 1 and 2; REP13, genotype ranking based on replicates 1 and 3.

In 2017 “OA1570-5-5Short” was identified as the highest yielding genotype whether based on all three replicates (Figure 10A) or on any two of them (Figure 10B). The correlation between genotypic means based on all three replicates and that based on any two replicates ranged from 0.955 to 0.961 and averaged 0.96 (*n* = 36).

**FIGURE 11:**
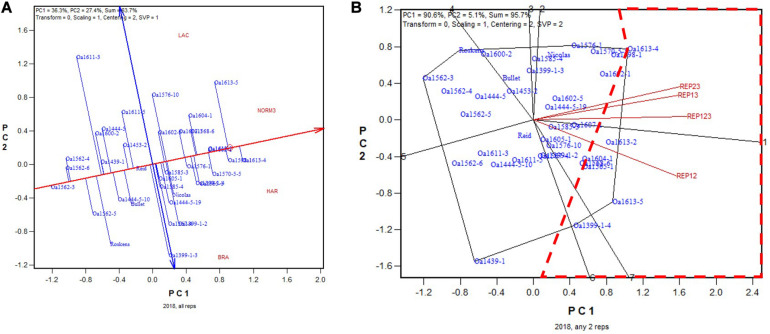
Biplots for the 2018 non-Ontario trials. **(A)** GGE biplot to show genotype ranking based on data from all three replicates, and **(B)** summary biplot to show the genotype rankings based on all three replicates and on any two of them. REP123, genotypic ranking based on all three replicates; REP12, genotype ranking based on replicates 1 and 2; REP13, genotype ranking based on replicates 1 and 3.

In 2018, the highest yielding genotype identified on all three replicates was OA1613-4 (Figure 11A). OA1613-4 was identified as the highest yielding in two of the three 2-rep combinations; for the other, REP12, it was identified as the 4th highest yielding (Figure 11B). The correlation between genotypic means based on all three replicates and that based on any two replicates ranged from 0.91 to 0.96 and averaged 0.93 (*n* = 36).

**FIGURE 12:**
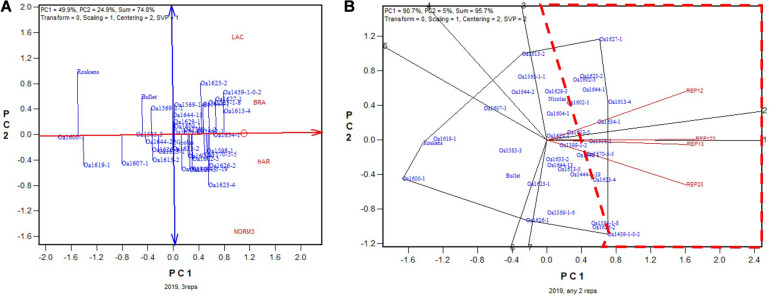
Biplots for the 2019 non-Ontario trials. **(A)** GGE biplot to show genotype ranking based on data from all three replicates, and **(B)** summary biplot to show the genotype rankings based on all three replicates and on any two of them. REP123, genotypic ranking based on all three replicates; REP12, genotype ranking based on replicates 1 and 2; REP13, genotype ranking based on replicates 1 and 3.

In 2019, the highest yielding genotype identified on all three replicates was OA1439-1-0-2, but it was placed closely to many other lines, meaning little difference from these higher yielding lines (Figure 12A). OA1439-1-0-2 was identified as the highest yielding in two of the three 2-rep combinations; for the other, REP12, it was identified to be the 7th highest yielding, after OA1627-1, OA1613-4, OA1634-1, etc. (Figure 12B). The correlation between genotypic means based on all three replicates and that based on any two replicates ranged from 093 to 0.94 and averaged 0.94 (*n* = 36). In summary, the empirical study showed that two replicates would have sufficed to identify the highest yielding genotypes for the 2015 to 2019 tests conducted at the non-Ontario test.

## Discussion

This study led to the quantitative definitions of “optimal replication” in crop variety trials on the single-trial basis and the multi-location trial basis. Comparing the two definitions led to the following understandings. First, due to the presence of GE or GL, single trial data analysis should be limited to data quality control and correction, and decision making should be based on cross-location analysis (Yan, 2014). Consequently, it is the definition on the cross-location basis that should be used to determine the optimum number of replicates. The estimation from the single-trial basis often leads to over estimation and should be avoided (Table 2 vs. Table 3). Second, the optimum number of replicates on the multi-location basis not only depends on the data quality in individual trials but also on the number of locations (Eqs 5 and 7). Fewer replicates may suffice with a greater number of test locations. This explains the experience that some breeding companies were successful in breeding superior cultivars by conducting yield trials at many locations with a single replicate (e.g., Forest Troyer, personal communication, 2003). Indeed, when the test is conducted in a sufficient number of locations, a single replication may suffice, particularly when supplemented with a proper experimental design such as an augmented design, a partially replicated design, blocking, or alpha lattices, and with a spatial analysis (Gilmour et al., 1997). Replication, randomization, and blocking (local control) are the three pillars of scientific experimentation (R.A. Fisher, from Street, 1990). Proper analysis, including spatial analysis within trials and GGE biplot analysis (Yan and Kang, 2002) or Factor Analytic analysis (Kelly et al., 2007) across trials, in accordance with the framework of dealing with genotype by environment interaction (Yan, 2016), may be regarded as the fourth pillar. Among these, randomization, local control, and data analysis should be maximally exploited as they involve little cost while replication should be minimized as it is costly. Third, although additional locations can compensate for reduced replicates, the opposite cannot be assumed. Excessive replicates cannot improve cross-location heritability beyond its maximum achievable level, which is determined only by the GL/G ratio and the number of locations (Eq. 6). Fourth, empirical results showed that two replicates were sufficient for the oat multi-location trials in Canada. This means that 50% (Ontario trials) to 33% (other provinces) of the plots can be saved without affecting the trial efficacy. The saved resources may be used in funding additional test locations or in improving the implementation of the trials at existing locations. Furthermore, the results for the Ontario trials suggested that a single replicate would suffice (Table 3). This provides a basis for reduced replication in future breeding trials. Nevertheless, for key trials that aim to provide data to the public, it is beneficial to use two replicates. Use of two replicates will allow assessment of the data quality in individual trials and will provide a buffer for potential loss of plots and data points.

According to Eq. 7, the estimated optimum number of replicates is proportional to the achievable cross-trial heritability. Therefore, the optimum number of replicates may be under-estimated if trials from multiple mega-environments are analyzed together, which would lead to a lower achievable cross-location heritability. It is, therefore, important to restrict the use of this equation within mega-environments. A good understanding of the target region through mega-environment analysis (Yan, 2015, 2019; Yan et al., 2020) is a prerequisite for conducting optimal replication analysis.

Although a formula was also derived for estimating the optimum number of replicates on the multi-year, multi-location basis (Eq. 12), it is not useful for systems in which decisions have to be made based on single-year data. It is useful, though, for systems that decisions are made based on data from a certain number of years. For example, the Quebec cereal crop committee requires 3 years of multi-location testing whereas the Prairie Grain Development Committee requires 2 years to decide if a genotype can be supported for registration. The optimum number of replicates estimated on a multi-year, multi-location basis can only be smaller than that on the single-year, multi-location basis. This represents a potential to reduce variety trial cost while maintaining trial efficacy.

Low trial heritability or the need for more replicates can result from (1) inconsistent soil conditions, (2) inconsistent weather conditions, (3) presence of any natural conditions that mask genotypic differences (e.g., an indiscriminative lodging or winterkill), (4) lack of certain natural conditions (e.g., a disease pressure) that are essential to reveal genotypic differences, (5) inconsistent management and handling, and/or (6) human mistakes. Under a scheme of reduced replication, one must spare no effort to improve, avoid, or address these factors by experimental design, implementation, and data analysis.

## Conclusion

Adequate replication in crop variety trials is important for reliable genotype evaluation. Based on the theory of quantitative genetics, equations were derived that quantitatively define optimal replication on the single-trial basis and the multi-location trial basis. The equation on single-trial basis often over-estimates the optimum number of replicates; it is the equation on multi-location trial basis that should be used. Applying the latter equation to the yield data from the 2015 to 2019 ORDC oat registration trials conducted both in Ontario and in other provinces of Canada led to the conclusion that a single replicate or two replicates would have sufficed in the current multi-location trial setup. This conclusion was confirmed by empirical comparison between genotypic ranking based on data from all replicates and that from any two of the replicates. This means that 33–50% of field plots could be saved without affecting the trial efficacy.

## Data Availability Statement

The original contributions presented in the study are included in the article/supplementary material, further inquiries can be directed to the corresponding author/s.

## Author Contributions

WY coordinated the ORDC oat registration test, developed the analytical methods, conducted the analyses, and wrote the manuscript.

## Conflict of Interest

The author declares that the research was conducted in the absence of any commercial or financial relationships that could be construed as a potential conflict of interest.

## References

[B1] Baxevanos D. Korpetis E. Irakli M. Tsialtas I. T (2017a). Evaluation of a durum wheat selection scheme under Mediterranean conditions: adjusting trial locations and replications. *Euphytica* 213:82 10.1007/s10681-017-1871-y

[B2] Baxevanos D. Tsialtas J. T. Vlachostergios D. Goulas C. (2017b). Optimum replications and locations for cotton cultivar trials under Mediterranean conditions. *J. Agricult. Sci.* 155 1553–1564. 10.1017/s0021859617000648

[B3] DeLacy I. H. Basford K. E. Cooper M. Bull J. K. McLaren C. G. (1996). “Analysis of multi-environment trials — A historical perspective,” in *Plant Adaptation and Crop Improvement*, eds Cooper M. Hammer G. L. (Wallingford: CAB International), 39–124.

[B4] Falconer D. S. (1989). *Introduction to Quantitative Genetics.* Essex: Longman Scientific and Technical.

[B5] George N. Lundy M. (2019). Quantifying genotype × environment effects in long-term common wheat yield trials from an agroecologically diverse production region. *Crop Sci.* 59 1960–1972. 10.2135/cropsci2019.01.0010

[B6] Gilmour A. R. Cullis B. R. Verbyla A. P. (1997). Accounting for natural and extraneous variation in the analysis of field experiments. *J. Agricult. Biol. Environ. Statist.* 2 269–293. 10.2307/1400446

[B7] Hanson W. D. Brim C. A. (1963). Optimal allocation of test material for two-stage testing with an application to evaluation of soybean lines. *Crop Sci.* 3 43–49. 10.2135/cropsci1963.0011183x000300010016x

[B8] Kelly A. M. Smith A. B. Eccleston J. A. Cullis B. R. (2007). The accuracy of varietal selection using factor analytic models for multi-environment plant breeding trials. *Crop Sci.* 47 1063–1070. 10.2135/cropsci2006.08.0540

[B9] Sprague G. F. Federer W. T. (1951). A comparison of variance components in corn yield trials. II. Error, year × variety, location × variety and variety components. *Agron J.* 43 535–541. 10.2134/agronj1951.00021962004300110003x

[B10] Street D. (1990). Fisher’s contributions to agricultural statistics. *Biometrics* 46 937–945. 10.2307/2532439

[B11] Swallow W. H. Wehner T. C. (1989). Optimum allocation of plots to years, seasons, locations, and replications, and its application to once-over-harvest cucumber trials. *Euphytica* 43 59–68. 10.1007/bf00037897

[B12] Woyann L. G. Zdziarski A. D. Zanella R. Rosa A. C. Conte J. Meira D. (2020). Optimal number of replications and test locations for soybean yield trials in Brazil. *Euphytica* 216:11.

[B13] Wricke G. Weber E. (1986). *Quantitative Genetics and Selection in Plant Breeding.* Berlin: Walter de Gruyter.

[B14] Xu N. Jin S. Q. Li J. (2016). Designing the national cotton variety trials regarding the number of replicates and number of test locations in China. *Acta Agron Sin.* 42 43–50 In Chinese with English abstract. 10.3724/sp.j.1006.2016.00043

[B15] Yan W. (2001). GGEbiplot—a Windows application for graphical analysis of multienvironment trial data and other types of two-way data. *Agron. J.* 93 1111–1118. 10.2134/agronj2001.9351111x

[B16] Yan W. (2014). *Crop Variety Trials: Data Management and Analysis.* Hoboken, NJ: John Wiley & Sons.

[B17] Yan W. (2015). Mega-environment analysis and test location evaluation based on unbalanced multiyear data. *Crop Sci.* 55 113–122. 10.2135/cropsci2014.03.0203

[B18] Yan W. (2016). Analysis and handling of G×E in a practical breeding program. *Crop Sci.* 56 2106–2118. 10.2135/cropsci2015.06.0336

[B19] Yan W. (2019). LG biplot: a graphical method for mega-environment investigation using existing crop variety trial data. *Sci. Rep.* 9:7130. 10.1038/s41598-019-43683-9 PMC6509248 31073232

[B20] Yan W. Frégeau-Reid J. Martin R. Pageau D. Mitchell-Fetch J. (2015). How many test locations and replications are needed in crop variety trials for a target region? *Euphytica* 202 361–372. 10.1007/s10681-014-1253-7

[B21] Yan W. Kang M. S. (2002). *GGE Biplot Analysis: A Graphical Tool for Breeders, Geneticists, and Agronomists*. Boca Raton, FL: CRC Press.

[B22] Yan W. Frégeau-Reid J. Martin R. Pageau D. Xue A. Jakubinek K. (2016). AAC Nicolas covered oat. *Canad. J. Plant Sci.* 97 132–134.

[B23] Yan W. Mitchell-Fetch J. Betties A. Nilsen K. Pageau D. deHaan B. (2020). Oat mega-environments in Canada. *Crop Sci.* 60 10.1002/csc2.20426

[B24] Yan W. Tinker N. A. Bekele W. A. Mitchell-Fetch J. Frégeau-Reid J. (2019). Theoretical unification and practical integration of conventional methods and genomic selection in plant breeding. *Crop. Breed. Genet. Genom.* 1:e190003 10.20900/cbgg20190003

[B25] Zhang Y. Xu N.-Y. Guo L.-L. Yang Z.-G. Zhang X.-Q. Yang X.-N. (2020). Optimization of test locations number and replication number in regional winter wheat variety trials in northern China. *Acta Agronom. Sin.* 46 1166–1173 In Chinese with English Abstract. 10.3724/sp.j.1006.2020.91069

[B26] Zhou M. Chihana A. Parfitt R. (2011). “Trends in variance components and optimum replications and crop-years for variety trials at Dwangwa sugar estate in Malawi,” in *Proceedings of the South African Sugar Technologists’ Association*, 84, Durban, 363–374.

